# Editorial: Vaccine clinical development in Latin America: achievements, challenges, and future perspectives

**DOI:** 10.3389/fmed.2024.1478343

**Published:** 2024-09-12

**Authors:** Rolando Ulloa-Gutierrez, Rodrigo DeAntonio

**Affiliations:** ^1^Servicio de Aislamiento, Hospital Nacional de Niños “Dr. Carlos Sáenz Herrera”, Caja Costarricense de Seguro Social (CCSS), San José, Costa Rica; ^2^Pediatrics - Infectious Diseases, Academia Nacional de Medicina de Costa Rica (ACANAMED), San José, Costa Rica; ^3^Pediatrics - Infectious Diseases, Universidad de Ciencias Médicas (UCIMED) e Instituto de Investigación en Ciencias Médicas (IICIMED), San José, Costa Rica; ^4^Cevaxin – Centro de Vacunación e Investigación, The Panama Clinic Complejo Pacific Center, Panama, Panama

**Keywords:** vaccination, Latin America, children, clinical development, risk factors, vaccine

Vaccines play a critical role in the prevention and control of infectious diseases and are also one of the most cost-effective health interventions. Collaborations between research institutions, investigators, hospitals, pharmaceutical companies, non-profit institutions, and government agencies, among others, have strengthened Latin American local capacity for vaccine clinical development in the last decades. However, challenges linked to infrastructure, regulatory harmonization, and other aspects persist.

This special Research Topic, “*Vaccine clinical development in Latin America: achievements, challenges, and future perspectives*,” aimed to present some of the vaccine research conducted in the region, the implementation of vaccination programs leading to disease control, the role of research to promote evidence-based decisions, and an overview of how the region is positioned in vaccine clinical development.

In the context of the SARS-CoV-2 pandemic, a rapid site readiness project launched in 2020 to perform COVID-19 vaccine efficacy trials demonstrated that Latin American sites were ready to conduct the studies (22 sites across seven countries were selected), although infrastructure and equipment were one of the biggest challenges and consumed most of the budget (1), confirming that the region has the potential to conduct trials not only in an emergency situation such as the pandemic but also on a regular basis.

In this Research Topic, regarding COVID-19, Díaz-Dinamarca et al. present a study conducted after the rollout of vaccines against SARS-CoV-2 in the context of the need for booster vaccinations against emerging virus variants, generating evidence to support the decision to promote heterologous vaccination in Chile.

The report by Torres et al., also from Chile, showed different strategies performed between interdisciplinary groups, such as the scientific community, government, and public and private institutions, to generate evidence to inform public health decisions during the pandemic in the country.

Although COVID-19 is one of the main public health topics addressed nowadays, the region also has a significant burden of other important infectious diseases, such as rotavirus and invasive pneumococcal disease. The results of mixed rotavirus vaccination schedules in Mexico are presented in the study by Macías-Parra et al., supporting the optimization of rotavirus vaccination programs for broader pediatric coverage. These results can be useful not only for the Latin American region but also worldwide.

For invasive pneumococcal disease, a prospective multicenter cohort of pediatric patients from 17 Colombian hospitals by Camacho-Moreno et al. reported an increase in the prevalence of serotypes 19A and 6C, which led to a change in the Colombian immunization schedule in 2022.

The introduction of COVID-19, pneumococcal, and rotavirus vaccines has significantly altered the landscape of disease pathogenesis for these infections in Latin America and globally. Rotavirus vaccines have reduced severe gastroenteritis in children, modifying the transmission. Likewise, pneumococcal vaccines have decreased the incidence of invasive disease by targeting multiple serotypes, leading to herd immunity and altering bacterial colonization patterns. COVID-19 vaccines have mitigated severe outcomes by reducing viral load and transmission, influencing the virus evolution including emergent new variants, and also influencing the host immune response. Together, these vaccines have reshaped pathogen behavior and immune responses, demonstrating the profound impact of immunization on disease dynamics.

As shown previously, vaccine research in Latin America impacts public health decisions and, therefore, the wellbeing of the population. We emphasize that more information could and should be available about this Research Topic, as the region has approximately 11,000 clinical trials per year (2). We encouraged investigators to publish their work, make it accessible to the scientific community, and, through initiatives like this, present the results that are making an impact not only in the region but also worldwide.
